# Supplementary figures and images for: Evaluation of a human mucosal tissue explant model for SARS-CoV-2 replication
Source: PLoS One. 2023 Sep 28;18(9):e0291146. doi: 10.1371/journal.pone.0291146 (PMC10538748; doi:10.1371/journal.pone.0291146)

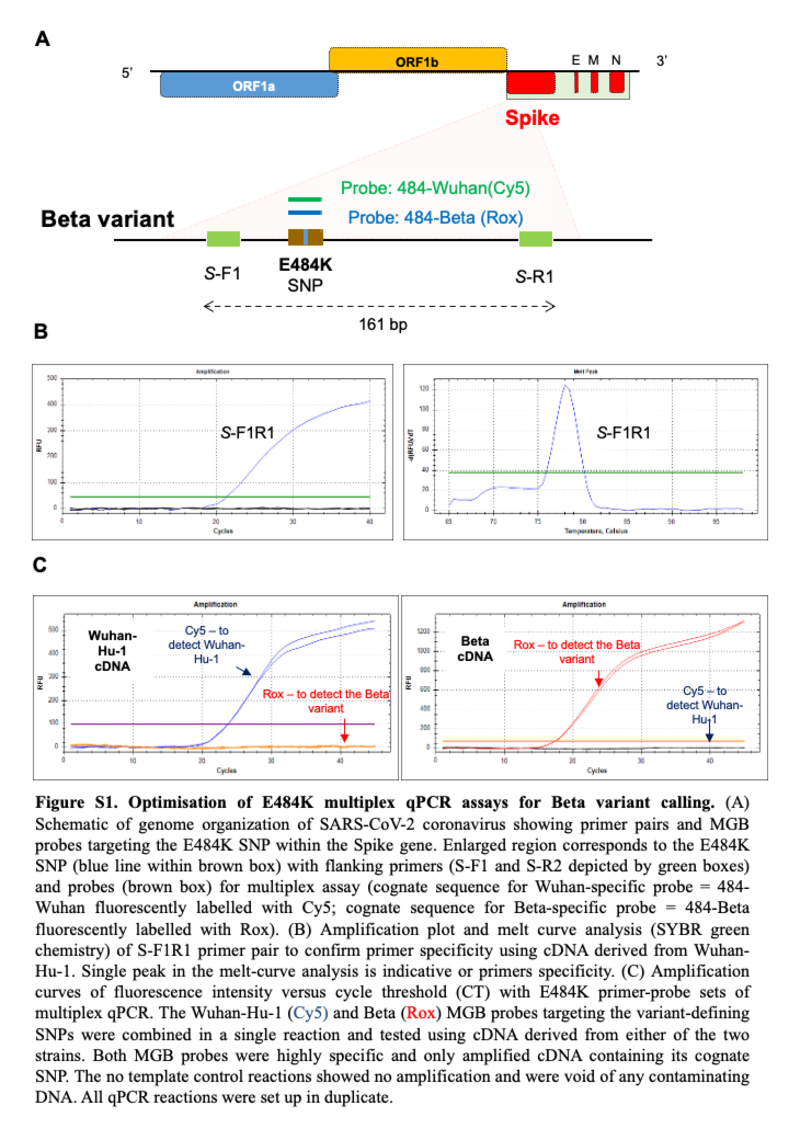

Supplement: S1 Fig — (A) Schematic of genome organization of SARS-CoV-2 coronavirus showing primer pairs and MGB probes targeting the E484K SNP within the Spike gene. Enlarged region corresponds to the E484K SNP (blue line within brown box) with flanking primers (S-F1 and S-R2 depicted by green boxes) and probes (brown box) for multiplex assay (cognate sequence for Wuhan-specific probe = 484-Wuhan fluorescently labelled with Cy5; cognate sequence for Beta-specific probe = 484-Beta fluorescently labelled with Rox). (B) Amplification plot and melt curve analysis (SYBR green chemistry) of S-F1R1 primer pair to confirm primer specificity using cDNA derived from Wuhan-Hu-1. A single peak in the melt-curve analysis is indicative of primer specificity. (C) Amplification curves of fluorescence intensity versus cycle threshold (CT) with E484K primer-probe sets of multiplex qPCR. The Wuhan-Hu-1 (Cy5) and Beta (Rox) MGB probes targeting the variant-defining SNPs were combined in a single reaction and tested using cDNA derived from either of the two strains. Both MGB probes were highly specific and only amplified cDNA containing its cognate SNP. The no template control reactions showed no amplification and were void of any contaminating DNA. All qPCR reactions were set up in duplicate. (TIF) [file pone.0291146.s001.tif]

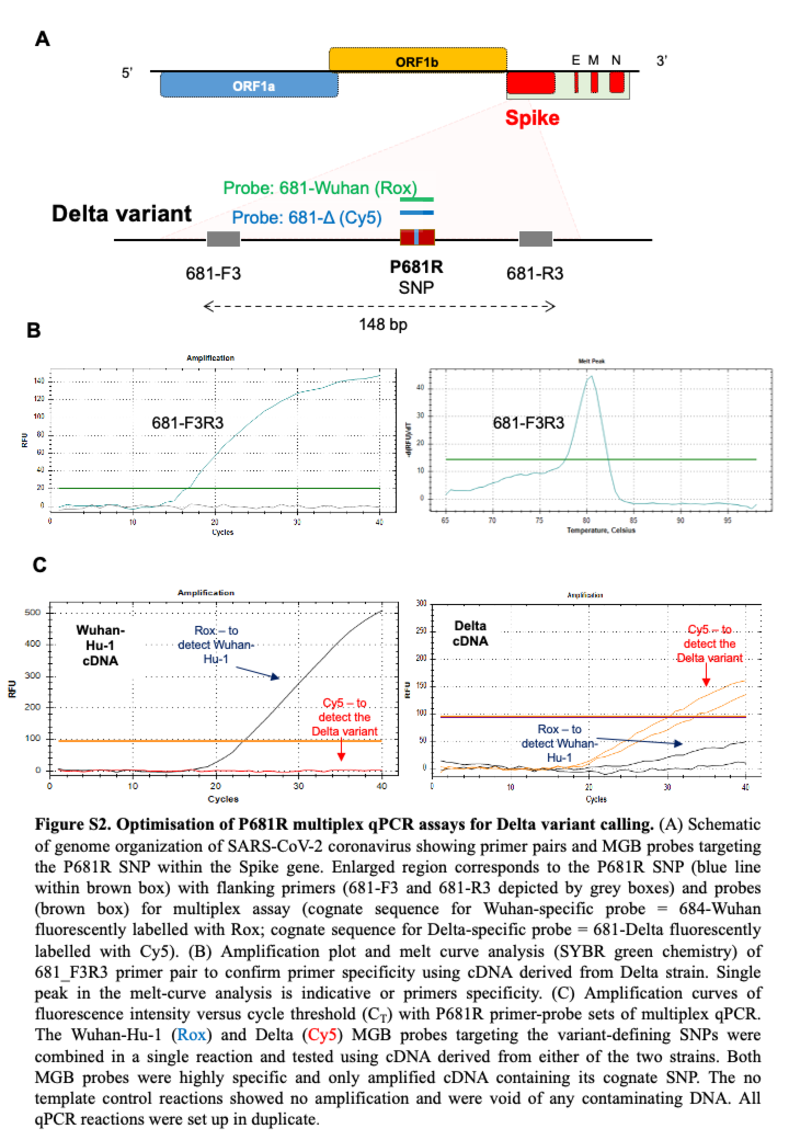

Supplement: S2 Fig — (A) Schematic of genome organization of SARS-CoV-2 coronavirus showing primer pairs and MGB probes targeting the P681R SNP within the Spike gene. Enlarged region corresponds to the P681R SNP (blue line within brown box) with flanking primers (681-F3 and 681-R3 depicted by grey boxes) and probes (brown box) for multiplex assay (cognate sequence for Wuhan-specific probe = 684-Wuhan fluorescently labelled with Rox; cognate sequence for Delta-specific probe = 681-Delta fluorescently labelled with Cy5). (B) Amplification plot and melt curve analysis (SYBR green chemistry) of 681_F3R3 primer pair to confirm primer specificity using cDNA derived from the Delta strain. A single peak in the melt-curve analysis is indicative of primer specificity. (C) Amplification curves of fluorescence intensity versus cycle threshold (CT) with P681R primer-probe sets of multiplex qPCR. The Wuhan-Hu-1 (Rox) and Delta (Cy5) MGB probes targeting the variant-defining SNPs were combined in a single reaction and tested using cDNA derived from either of the two strains. Both MGB probes were highly specific and only amplified cDNA containing its cognate SNP. The no template control reactions showed no amplification and were void of any contaminating DNA. All qPCR reactions were set up in duplicate. (TIF) [file pone.0291146.s002.tif]

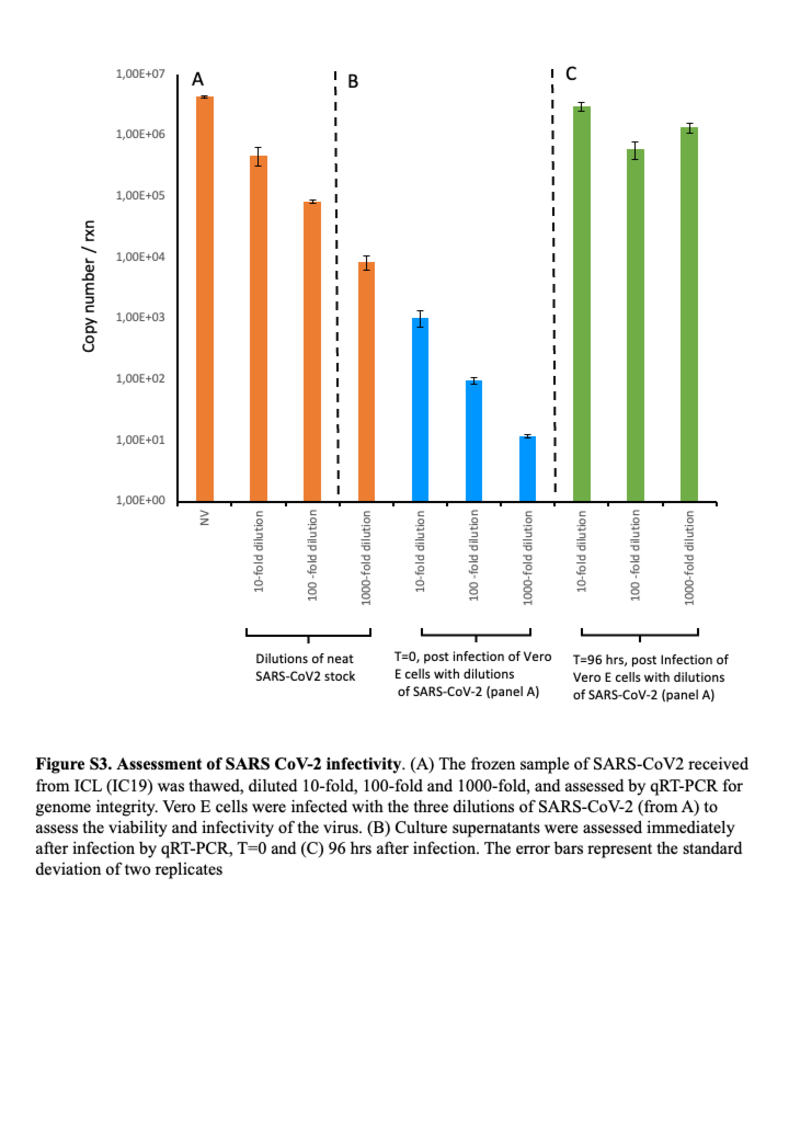

Supplement: S3 Fig — (A) The frozen sample of SARS-CoV2 received from ICL (IC19) was thawed, diluted 10-fold, 100-fold and 1000-fold, and assessed by qRT-PCR for genome integrity. Vero E cells were infected with the three dilutions of SARS-CoV-2 (from A) to assess the viability and infectivity of the virus. (B) Culture supernatants were assessed immediately after infection by qRT-PCR, T = 0 and (C) 96 hrs after infection. The error bars represent the standard deviation of two replicates. (TIF) [file pone.0291146.s003.tif]

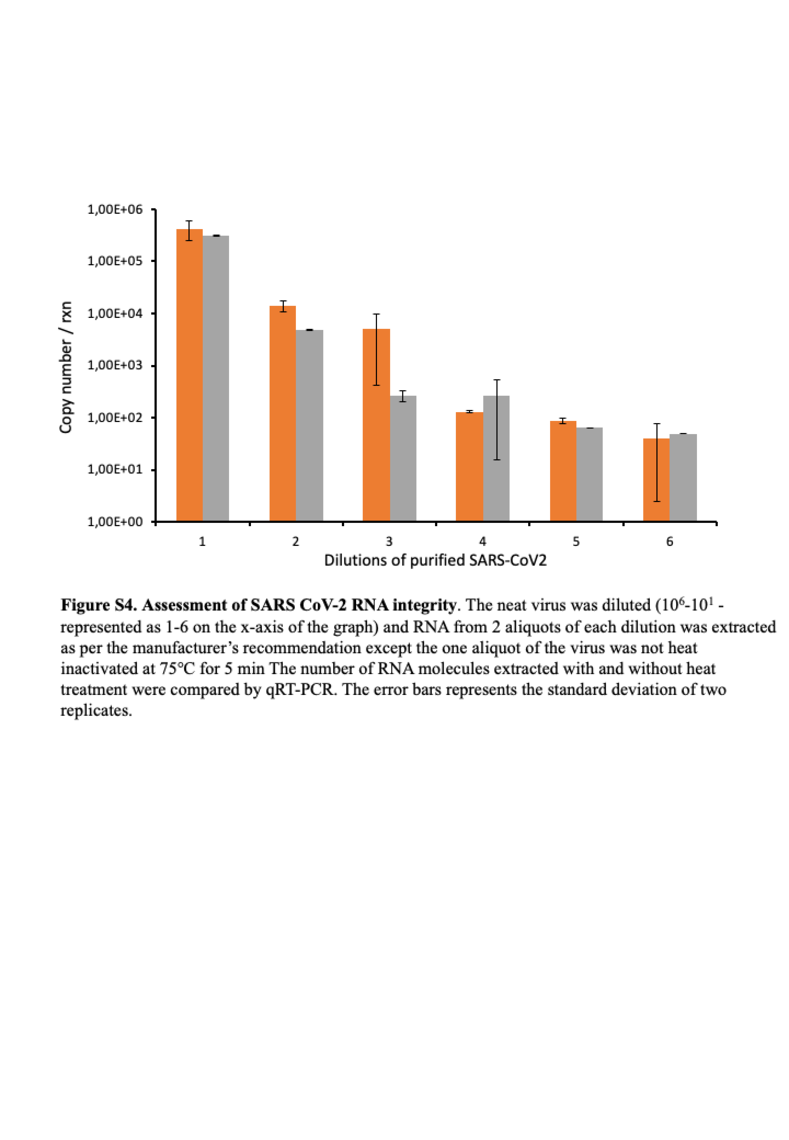

Supplement: S4 Fig — The neat virus was diluted (106−101—represented as 1–6 on the x-axis of the graph) and RNA from 2 aliquots of each dilution was extracted as per the manufacturer’s recommendation except the one aliquot of the virus was not heat inactivated at 75°C for 5 min The number of RNA molecules extracted with and without heat treatment were compared by qRT-PCR. The error bars represents the standard deviation of two replicates. (TIF) [file pone.0291146.s004.tif]

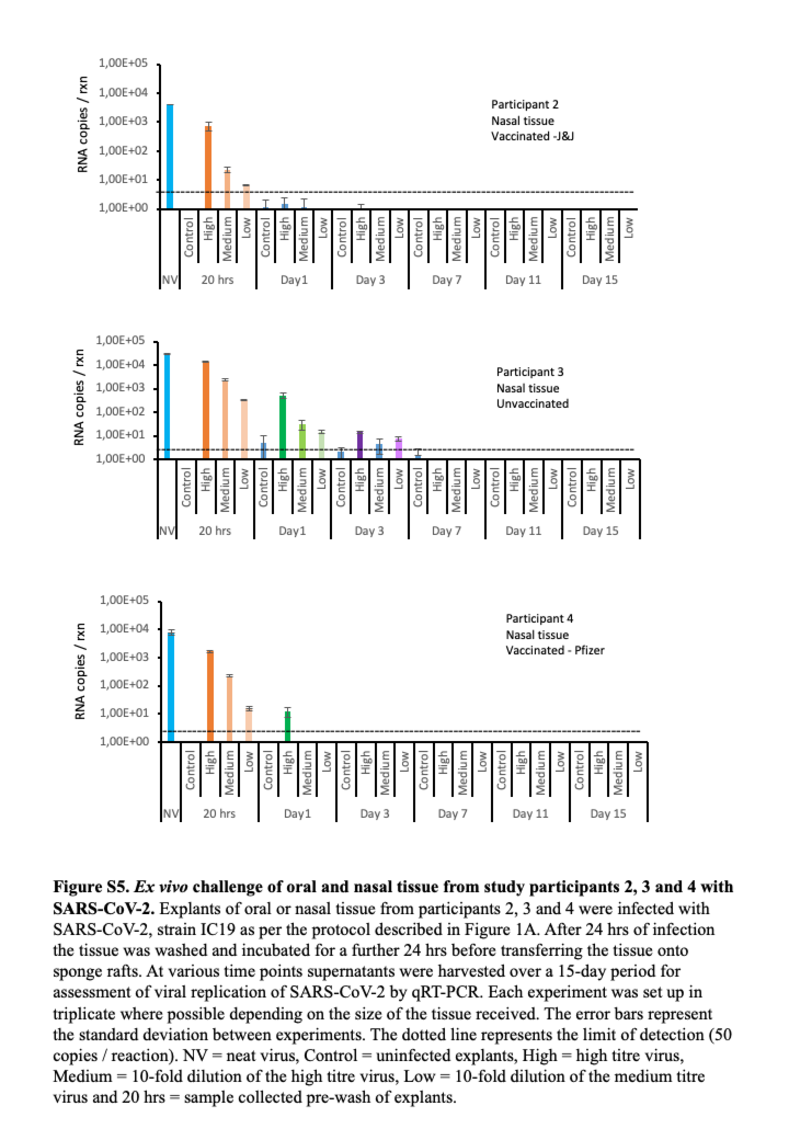

Supplement: S5 Fig — Explants of oral or nasal tissue from participants 2, 3 and 4 were infected with SARS-CoV-2, strain IC19 as per the protocol described in Fig 1A. After 24 hrs of infection the tissue was washed and incubated for a further 24 hrs before transferring the tissue onto sponge rafts. At various time points supernatants were harvested over a 15-day period for assessment of viral replication of SARS-CoV-2 by qRT-PCR. Each experiment was set up in triplicate where possible depending on the size of the tissue received. The error bars represent the standard deviation between experiments. The dotted line represents the limit of detection (50 copies / reaction). NV = neat virus, Control = uninfected explants, High = high titre virus, Medium = 10-fold dilution of the high titre virus, Low = 10-fold dilution of the medium titre virus and 20 hrs = sample collected pre-wash of explants. (TIF) [file pone.0291146.s005.tif]

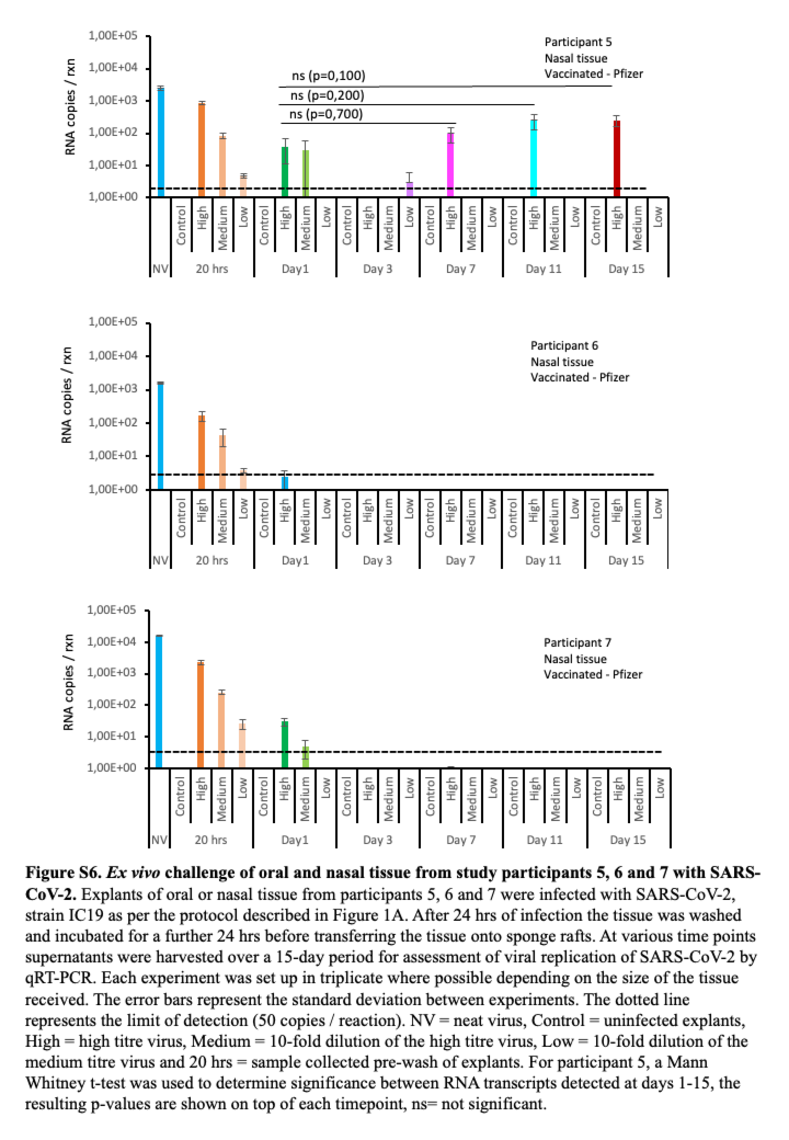

Supplement: S6 Fig — Explants of oral or nasal tissue from participants 5, 6 and 7 were infected with SARS-CoV-2, strain IC19 as per the protocol described in Fig 1A. After 24 hrs of infection the tissue was washed and incubated for a further 24 hrs before transferring the tissue onto sponge rafts. At various time points supernatants were harvested over a 15-day period for assessment of viral replication of SARS-CoV-2 by qRT-PCR. Each experiment was set up in triplicate where possible depending on the size of the tissue received. The error bars represent the standard deviation between experiments. The dotted line represents the limit of detection (50 copies / reaction). NV = neat virus, Control = uninfected explants, High = high titre virus, Medium = 10-fold dilution of the high titre virus, Low = 10-fold dilution of the medium titre virus and 20 hrs = sample collected pre-wash of explants. For participant 5, a Mann Whitney t-test was used to determine significance between RNA transcripts detected at days 1–15, the resulting p-values are shown on top of each timepoint, ns = not significant. (TIF) [file pone.0291146.s006.tif]

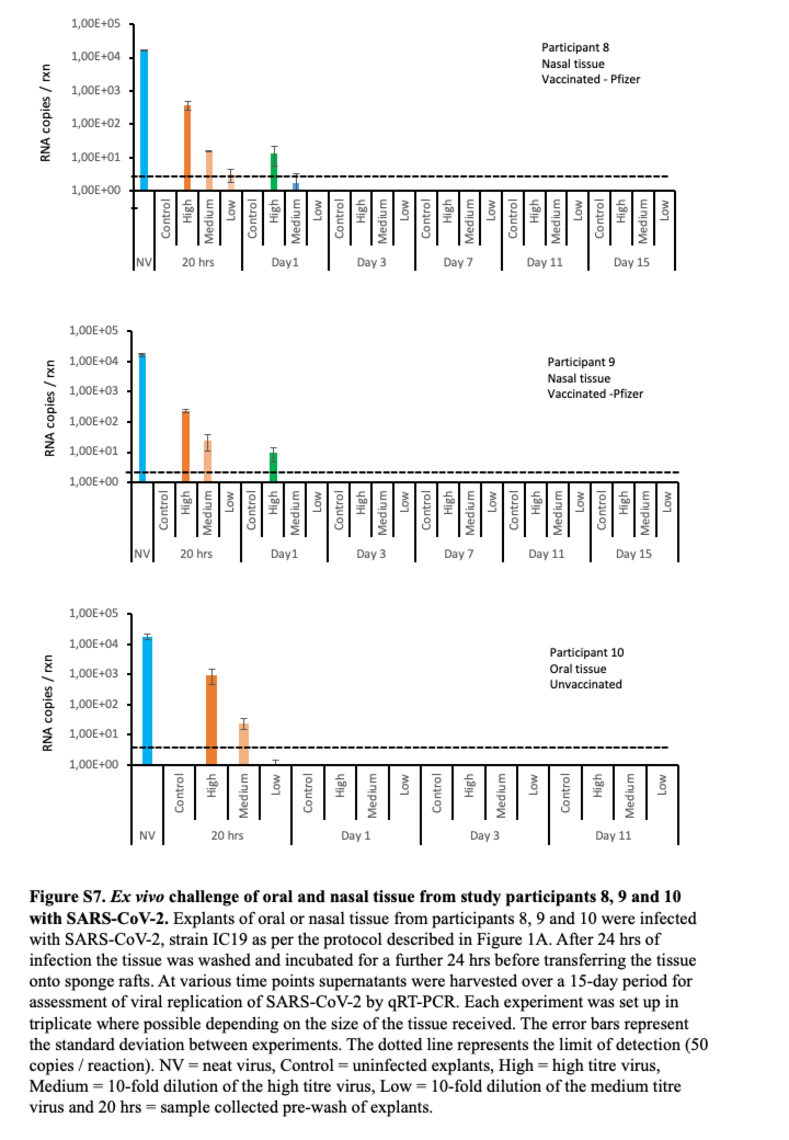

Supplement: S7 Fig — Explants of oral or nasal tissue from participants 8, 9 and 10 were infected with SARS-CoV-2, strain IC19 as per the protocol described in Fig 1A. After 24 hrs of infection the tissue was washed and incubated for a further 24 hrs before transferring the tissue onto sponge rafts. At various time points supernatants were harvested over a 15-day period for assessment of viral replication of SARS-CoV-2 by qRT-PCR. Each experiment was set up in triplicate where possible depending on the size of the tissue received. The error bars represent the standard deviation between experiments. The dotted line represents the limit of detection (50 copies / reaction). NV = neat virus, Control = uninfected explants, High = high titre virus, Medium = 10-fold dilution of the high titre virus, Low = 10-fold dilution of the medium titre virus and 20 hrs = sample collected pre-wash of explants. (TIF) [file pone.0291146.s007.tif]

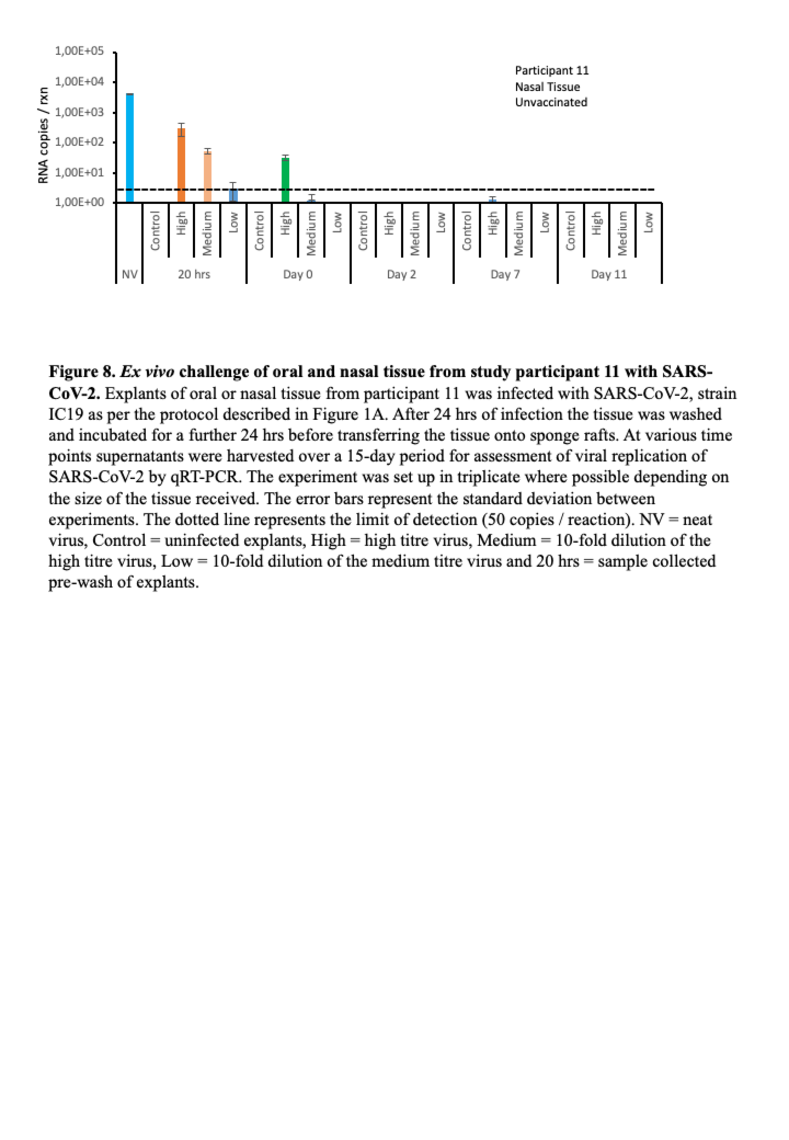

Supplement: S8 Fig — Explants of oral or nasal tissue from participant 11 was infected with SARS-CoV-2, strain IC19 as per the protocol described in Fig 1A. After 24 hrs of infection the tissue was washed and incubated for a further 24 hrs before transferring the tissue onto sponge rafts. At various time points supernatants were harvested over a 15-day period for assessment of viral replication of SARS-CoV-2 by qRT-PCR. The experiment was set up in triplicate where possible depending on the size of the tissue received. The error bars represent the standard deviation between experiments. The dotted line represents the limit of detection (50 copies / reaction). NV = neat virus, Control = uninfected explants, High = high titre virus, Medium = 10-fold dilution of the high titre virus, Low = 10-fold dilution of the medium titre virus and 20 hrs = sample collected pre-wash of explants. (TIF) [file pone.0291146.s008.tif]

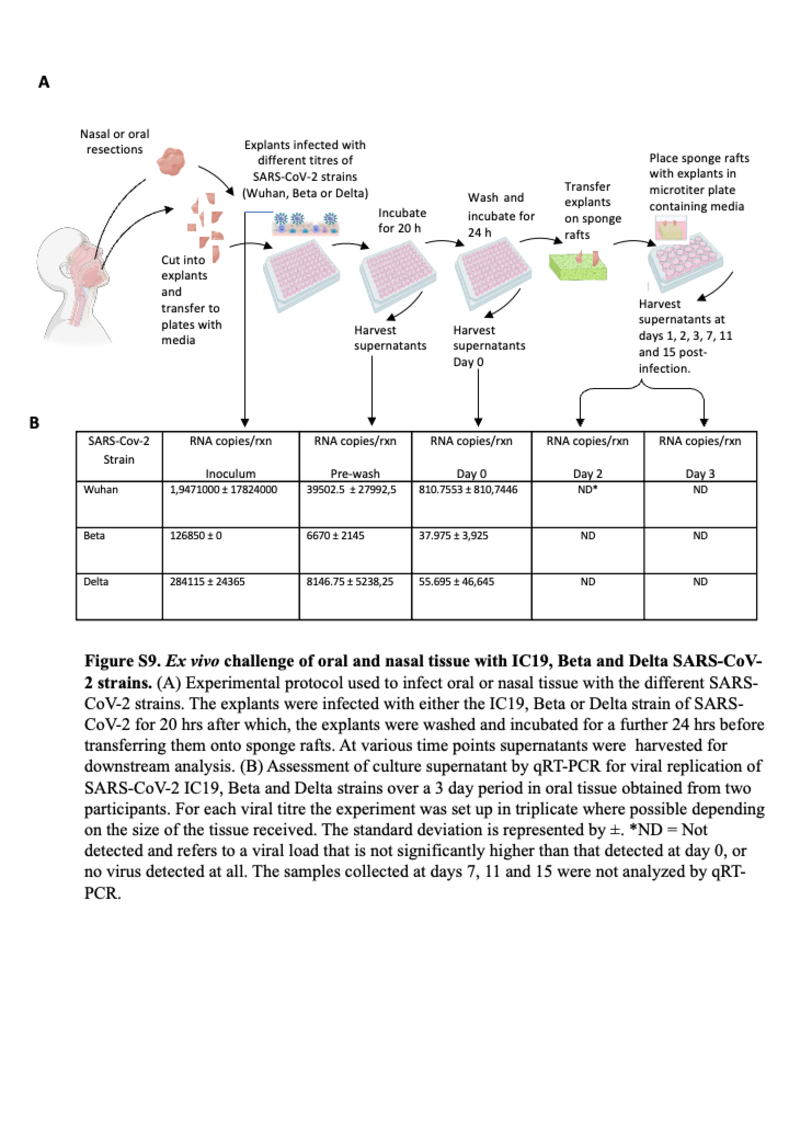

Supplement: S9 Fig — (A) Experimental protocol used to infect oral or nasal tissue with the different SARS-CoV-2 strains. The explants were infected with either the IC19, Beta or Delta strain of SARS-CoV-2 for 20 hrs after which, the explants were washed and incubated for a further 24 hrs before transferring them onto sponge rafts. At various time points supernatants were harvested for downstream analysis. (B) Assessment of culture supernatant by qRT-PCR for viral replication of SARS-CoV-2 IC19, Beta and Delta strains over a 3 day period in oral tissue obtained from two participants. For each viral titre the experiment was set up in triplicate where possible depending on the size of the tissue received. The standard deviation is represented by ±. *ND = Not detected and refers to a viral load that is not significantly higher than that detected at day 0, or no virus detected at all. The samples collected at days 7, 11 and 15 were not analyzed by qRT-PCR. (TIF) [file pone.0291146.s009.tif]

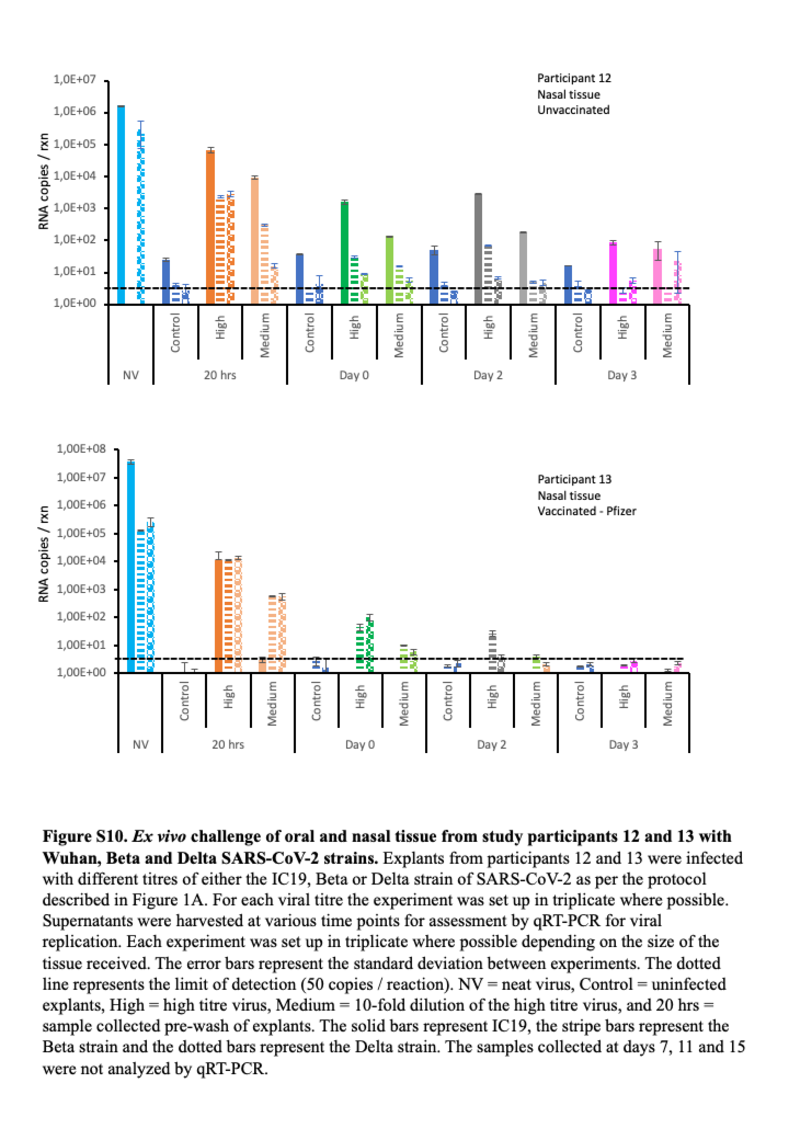

Supplement: S10 Fig — Explants from participants 12 and 13 were infected with different titres of either the IC19, Beta or Delta strain of SARS-CoV-2 as per the protocol described in Fig 1A. For each viral titre the experiment was set up in triplicate where possible. Supernatants were harvested at various time points for assessment by qRT-PCR for viral replication. Each experiment was set up in triplicate where possible depending on the size of the tissue received. The error bars represent the standard deviation between experiments. The dotted line represents the limit of detection (50 copies / reaction). NV = neat virus, Control = uninfected explants, High = high titre virus, Medium = 10-fold dilution of the high titre virus, and 20 hrs = sample collected pre-wash of explants. The solid bars represent IC19, the stripe bars represent the Beta strain and the dotted bars represent the Delta strain. The samples collected at days 7, 11 and 15 were not analyzed by qRT-PCR. (TIF) [file pone.0291146.s010.tif]

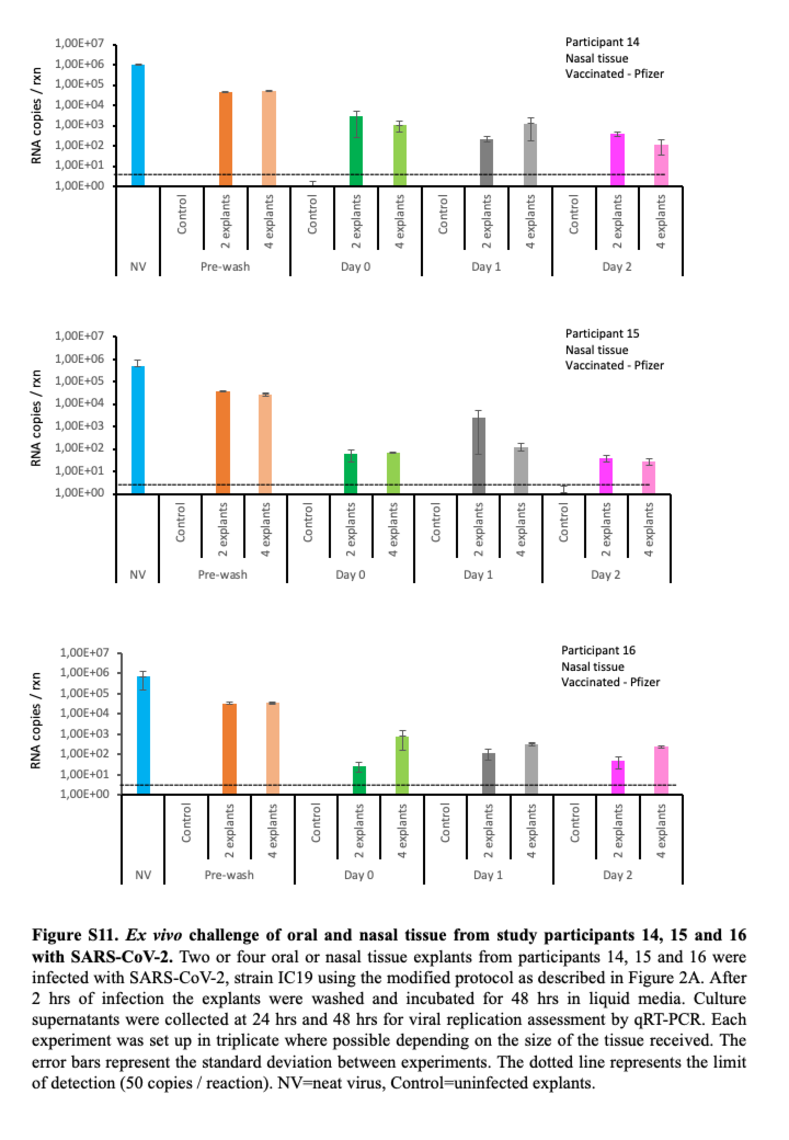

Supplement: S11 Fig — Two or four oral or nasal tissue explants from participants 14, 15 and 16 were infected with SARS-CoV-2, strain IC19 using the modified protocol as described in Fig 2A. After 2 hrs of infection the explants were washed and incubated for 48 hrs in liquid media. Culture supernatants were collected at 24 hrs and 48 hrs for viral replication assessment by qRT-PCR. Each experiment was set up in triplicate where possible depending on the size of the tissue received. The error bars represent the standard deviation between experiments. The dotted line represents the limit of detection (50 copies / reaction). NV = neat virus, Control = uninfected explants. (TIF) [file pone.0291146.s011.tif]

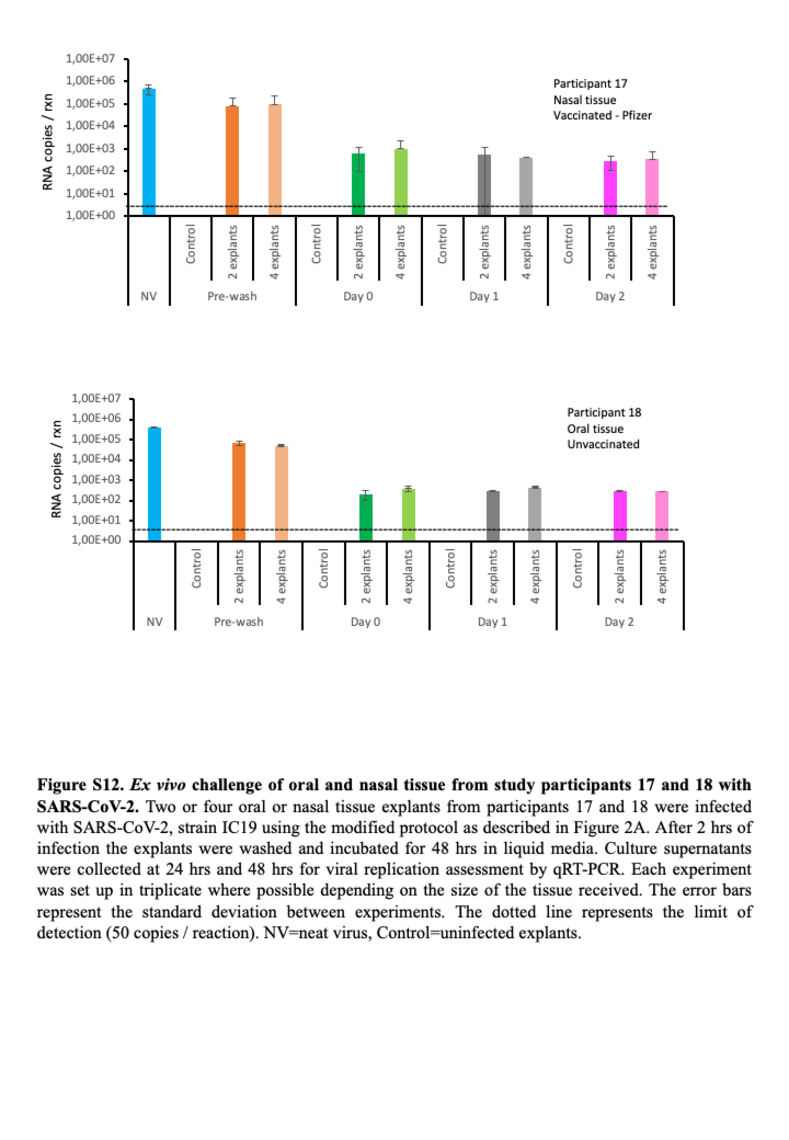

Supplement: S12 Fig — Two or four oral or nasal tissue explants from participants 17 and 18 were infected with SARS-CoV-2, strain IC19 using the modified protocol as described in Fig 2A. After 2 hrs of infection the explants were washed and incubated for 48 hrs in liquid media. Culture supernatants were collected at 24 hrs and 48 hrs for viral replication assessment by qRT-PCR. Each experiment was set up in triplicate where possible depending on the size of the tissue received. The error bars represent the standard deviation between experiments. The dotted line represents the limit of detection (50 copies / reaction). NV = neat virus, Control = uninfected explants. (TIF) [file pone.0291146.s012.tif]
