# Supplementary material for: Evaluation of a human mucosal tissue explant model for SARS-CoV-2 replication
Source: PLoS One. 2023 Sep 28;18(9):e0291146. doi: 10.1371/journal.pone.0291146 (PMC10538748; doi:10.1371/journal.pone.0291146)
Supplement: S1 Table — (DOCX) [file pone.0291146.s013.docx]

**S1 Table. Primers and probe sequences (with modifications) for variant calling and cDNA synthesis of Wuhan, Beta and Delta lineages of SARS-CoV-2.**

| **Assay/SNP target** | **Variant calling** | **Oligonucleotide** | **Sequence**  **5’ or 3’ modification indicated on probe (P)** | **T_m_ & amplicon size** | **Final concentration in assay** |
| --- | --- | --- | --- | --- | --- |
| **E-gene** | N/A | ES-F  ES-R | 5’ACAGGTACGTTAATAGTTAATAGCGT-3’  5’-ATATTGCAGCAGTACGCACACA-3’ | 59°C / 125 bp | 375 nM  375 nM |
| **E484K** | Beta | S-F1  S-R1  P:484_Wuhan  P:484_Beta | 5’-CAACTGAAATCTATCAGGCC-3’  5‘-TGCTGGTGCATGTAGAAGTT-3’  5’-(Cy5)-GTAATGGTGTTGAAGGTT-(MGB)-3’  5’-(Rox)-TGTAATGGTGTTAAAGGTTT-(MGB)-3’ | 60°C / 161 bp | 300 nM  300 nM  150 nM  150 nM |
| **P681R** | Delta | 681_F3  681_R3  P:681_Wuhan  P:681_Delta | 5’- CATATGAGTGTGACATACCC-3’  5’- GAGTAAGCAACTGAATTTTC-3’  ‘5’-(Rox)-CCCGCCGAGGAGAATT-(MGB)-3’  5’-(Cy5)-CCGCCGACGAGAATTA-(MGB)-3’ | 60°C / 148 bp | 300 nM  300 nM  150 nM  150 nM |
| **N/A #** | N/A | Spike_cDNA | 5’- GATTAGCAGAAGCTCTGATT – 3’ | N/A | 2.5 µM |

**#** Used specifically for the synthesis of complementary cDNA with Superscript IV reverse transcriptase (Thermofisher Scientific).
