# Supplementary material for: Evaluation of a human mucosal tissue explant model for SARS-CoV-2 replication
Source: PLoS One. 2023 Sep 28;18(9):e0291146. doi: 10.1371/journal.pone.0291146 (PMC10538748; doi:10.1371/journal.pone.0291146)
Supplement: Attachment — Submitted filename: Response to reviewers.docx [file pone.0291146.s017.docx]

**DST/NRF Centre of Excellence for Biomedical TB Research**

University of the Witwatersrand

National Health Laboratory Service

Johannesburg, South Africa

Tel: +27 11 489 9376

Fax: +27 11 489 9397

<http://www.wits.ac.za/cbtbr>

twitter.com/CBTBRwits

facebook.com/CBTBR

Date: 3^rd^ August 2023

**Attention:** Editorial Office

PLOS ONE

**Re: Revised submission of an original research manuscript**

To the Editor-in-Chief

Please find appended our response to reviewers comments for the revised submission of our manuscript entitled ‘Evaluation of a human mucosal tissue explant model for SARS-CoV-2 replication’ PONE-D-23-15626

**Response to reviewers comments**

PONE-D-23-15626

Evaluation of a human mucosal tissue explant model for SARS-CoV-2 replication

PLOS ONE

We would like to thank the reviewers and editors for their careful consideration of our work. We are also grateful for the insights and suggestions to improve the manuscript. A point-by-point response is provided below.

1. Please ensure that your manuscript meets PLOS ONE's style requirements, including those for file naming. The PLOS ONE style templates can be found at 
<https://journals.plos.org/plosone/s/file?id=wjVg/PLOSOne_formatting_sample_main_body.pdf> and 
<https://journals.plos.org/plosone/s/file?id=ba62/PLOSOne_formatting_sample_title_authors_affiliations.pdf>

**Author response:** Our files meet the requirements.

2. We note that the grant information you provided in the ‘Funding Information’ and ‘Financial Disclosure’ sections do not match.

When you resubmit, please ensure that you provide the correct grant numbers for the awards you received for your study in the ‘Funding Information’ section.

**Author Response:** We have addressed this.

3. Please expand the acronym “EDCTP” (as indicated in your financial disclosure) so that it states the name of your funders in full.
This information should be included in your cover letter; we will change the online submission form on your behalf.

**Author Response:** This has been addressed in the Manuscript

EDCTP represents, The European and Developing Countries Clinical Trials Partnership.

4. We note that you have included the phrase “data not shown” in your manuscript. Unfortunately, this does not meet our data sharing requirements. PLOS does not permit references to inaccessible data. We require that authors provide all relevant data within the paper, Supporting Information files, or in an acceptable, public repository. Please add a citation to support this phrase or upload the data that corresponds with these findings to a stable repository (such as Figshare or Dryad) and provide and URLs, DOIs, or accession numbers that may be used to access these data. Or, if the data are not a core part of the research being presented in your study, we ask that you remove the phrase that refers to these data.

**Author Response:** This term has been removed and all the data are now provided in the supplementary information.

5. We note that Figures 1 and 2 in your submission contain copyrighted images. All PLOS content is published under the Creative Commons Attribution License (CC BY 4.0), which means that the manuscript, images, and Supporting Information files will be freely available online, and any third party is permitted to access, download, copy, distribute, and use these materials in any way, even commercially, with proper attribution. For more information, see our copyright guidelines: <http://journals.plos.org/plosone/s/licenses-and-copyright>.
 
We require you to either (1) present written permission from the copyright holder to publish these figures specifically under the CC BY 4.0 license, or (2) remove the figures from your submission:
 
a. You may seek permission from the original copyright holder of Figures 1 and 2 to publish the content specifically under the CC BY 4.0 license. 
 
We recommend that you contact the original copyright holder with the Content Permission Form (<http://journals.plos.org/plosone/s/file?id=7c09/content-permission-form.pdf>) and the following text:
“I request permission for the open-access journal PLOS ONE to publish XXX under the Creative Commons Attribution License (CCAL) CC BY 4.0 (<http://creativecommons.org/licenses/by/4.0/>). Please be aware that this license allows unrestricted use and distribution, even commercially, by third parties. Please reply and provide explicit written permission to publish XXX under a CC BY license and complete the attached form.”
 
Please upload the completed Content Permission Form or other proof of granted permissions as an "Other" file with your submission. 
 
In the figure caption of the copyrighted figure, please include the following text: “Reprinted from [ref] under a CC BY license, with permission from [name of publisher], original copyright [original copyright year].”
 
b. If you are unable to obtain permission from the original copyright holder to publish these figures under the CC BY 4.0 license or if the copyright holder’s requirements are incompatible with the CC BY 4.0 license, please either i) remove the figure or ii) supply a replacement figure that complies with the CC BY 4.0 license. Please check copyright information on all replacement figures and update the figure caption with source information. If applicable, please specify in the figure caption text when a figure is similar but not identical to the original image and is therefore for illustrative purposes only.

**Author Response:** We generated our figures using Biorender. We have credited them for this in the figure legends. There are no other copyright issues.

**Reviewers' comments:**

Reviewer #1:

The study addresses the issue to disease modeling in Covid infection with the ultimate goal to develop a reliable, reproducible and clinically relevant model of virus entry, replication and biological effects. Such a model could be useful in studying viral characteristics such as entry and life-cycle, pathogenic features but most importantly evaluating the efficacy of novel drugs. Disease modeling using human samples is of particular importance in the absence of suitable animal models for SARS-CoV-2 infection. Among well accepted approaches in the literature are explant cultures of target tissues for the virus, primarily lung tissue. Since the virus can enter the body via and be detected in significant amounts in the nasal and oral cavities, the investigators have used for the explant experiments residual oral and nasal tissue obtained from 18 individuals undergoing elective maxillofacial surgery.

**Author Response**: We thank the reviewer for assessing the manuscript and for the useful comments to strengthen the manuscript.

It is not clear the type of epithelium present in those tissues and perhaps a more detailed description, some representative H&E stained pictures or adding a patient table might have contributed to a better understanding of the experimental approach.

**Author Response:** We agree that H&E staining of the tissue would be useful but it was not carried out on the tissue as we do not have the facility to carry out such experiments. At this point, we have indicate the the source of the tissue, oral or nasal tissue for each participant on the individual graphs.

We have inserted a patient demographics table, this is now Table 1.

The tissue was transferred within one hour of its harvesting to the lab which is an advantage for the overall tissue survival. It is not mentioned whether the tissues were frozen for the experiment or used as fresh material.

The tissue was used as fresh material, immediately as it was received in the lab. We have included text “Once received at the laboratory the explant tissue was prepared immediately for in vitro infection challenge, under the materials and methods section.

The investigators quite appropriately used titration experiments for the viral input. Overall they should be commented for presenting in detail and in a comprehensive way (supported by diagrammatic study overview for each experiment) their different approaches to establish viral replication in an explant system and providing adequate rational for pursuing several protocol modifications. Appropriate controls have been included. Unfortunately, the team was not able to demonstrate significant viral replication in their system and in our opinion, publication of negative data should be encouraged.

**Author Response**: We thank the reviewer for appreciating our work and for encouraging publication of the data. This represented 2 years of careful work by clinical and laboratory teams. We agree that there is merit for the results to feature in the published literature.

However, as it is currently structured the manuscript appears rather descriptive, particularly data presented in Fig 3.

**Author Response:** We agree with the reviewer. The data presented in Figure 3 are limited and require optimization, which currently is not possible due to limited sample volume. As suggested by the reviewer we have decided to remove these data from the manuscript.

For example, one of the strengths of the explant approach is that is allows studying patient heterogeneity. For example, it would be of interest in Fig 1 the results to be presented for each patient individually (a panel of 10 datasets) and also clarify whether the tissue was of oral or nasal origin.

**Author Response**: This has been included in the Supplementary Information as Figure S5

Same is true for Fig 2. Furthermore, data analysis should be performed in relation to information about tissue histology and additional patient characteristics that might potentially impact tissue survival and suitability for explant infection.

**Author Response**: We agree with the reviewer that histology is important however, we did not have the facility or expertise to do this. We have included the individual graphs in the Supplementary Information, Figure S8. Patient characteristics are reported in Table 1.

It has been previously reported that the survival of epithelial cells from the gastrointestinal tract is substantially declining after 24 hours in culture. Which exact type of cells are the investigators aiming at infecting with the virus and under which kinetics assumptions?

**Author Response:** We did not characterize the precise cellular of the mucosal tissue. As per previous publications listed below, we used fresh mucosal tissue, without prior characterization of cellular components:

(1) Analysis of Severe Acute Respiratory Syndrome 2 Replication in Explant Cultures of the Human Upper Respiratory Tract Reveals Broad Tissue Tropism of Wild-Type and B.1.1.7 Variant Viruses. J Infect Dis. 2021;224(12):2020-4. doi: 10.1093/infdis/jiab523. PubMed PMID: 34651643; PubMed Central PMCID: PMCPMC8524635

(2) Human Nasal and Lung Tissues Infected Ex Vivo with SARS-CoV-2 Provide Insights into Differential Tissue-Specific and Virus-Specific Innate Immune Responses in the Upper and Lower Respiratory Tract. J Virol. 2021;95(14):e0013021. Epub 20210624. doi: 10.1128/JVI.00130-21. PubMed PMID: 33893170; PubMed Central PMCID: PMCPMC8223920

The specific type of tissue used for each participant is shown in the graphs in the Supplementary Information.

In Fig 3 a diagram of the experimental protocol should be added, as in Fig 1-2, in order to facilitate the interpretation of the results. It would be helpful if the authors could add a working hypothesis for the experiment described in Fig 3. Are the neutralizing antibodies originating from residual blood in the tissue or are produced de novo from infiltrating B-cells?

As mentioned by the reviewer, these data are puzzling from a technical point of view as we obtained plaques with or without heat treatment. Currently, we will not be able to address these concerns as we do not have sufficient tissues or supernatants. As suggested, we have decided to remove these experiments from the manuscript as they do not add value to the manuscript.

In its current form, data presented in Fig 3 is rather “anecdotal” since only two patients have been profiled. Again, in order to gain any meaningful conclusions, all available post-wash samples should be tested using the same methodology and data from all available patients should be presented in Fig 3.

**Author Response**: We agree with the reviewer. However, the sample size for each protocol was small and also the harvested culture supernatant was limited. Our Institutional BioSafety Committee provided clearance for this work based on only small volumes being used for the studies. Hence, we do not have much material to conduct further tests. We also do not have a good representation of vaccinated and unvaccinated participants for optimizing these experiments and therefore as suggested below, we have decided to remove these data from the manuscript.

In Fig 3, what is the NC (non-culture supernatant) control? Is it culture media with serum?

**Author Response**: The abbreviation is CS which represents culture supernatant. CS is the media containing the tissue explant that is harvested before infection and at various time points after infection. This figure has been removed from the manuscript, hence this information is irrelevant.

Data presented in Fig 3 is puzzling from a technical point of view as well. For instance, in vaccinated participant 1, why in NC control incubation at 65oC enhanced viral replication and at the same time reduced viral replication in the CS? The plaque assay should be run at least in triplicate for each patient due to method variability. In vaccinated participant 2, a similar concern was raided: There is apparent viral replication enhancement under all conditions, irrespectively of explant secreted factors. Those concerns should be adequately addressed by the investigators or if not possible due to technical reasons, the neutralization assessment experiments should be taken out of this manuscript till the methodology is working properly based on positive and negative controls.

**Author Response**: We agree with the reviewer. This experiment definitely needs further optimization and replication for meaningful interpretation. However, due to the small participant size and limited sample volume we are unable to carry out the plaque assay with different experimental conditions and in triplicate. At this stage we will not be able to address all the technical aspects in triplicate together with positive and negative controls and agree with the reviewer that these data are not adding to the manuscript. Therefore, we have removed these data from the manuscript as per the reviewers suggestion.

Discussion needs to be extended and specifically address how the methodology used in this study (Fig1 &2) compares to previous publications using nasal explants (REF 11,13).

**Author Response**: This has been done.

Reviewer #2: The authors have conducted a clinically relevant study with translational potential. The study design is appropriate for the study question and the scientific weakness has been properly identified and mentioned. The data analysis and interpretation and discussion of the outcome is scientifically sound.

**Author Response**: We thank the reviewer for assessing the manuscript and for the useful comments to strengthen the manuscript.

The authors have not mentioned what precautions were taken in the lab or during the experiments to prevent transmission of the infection to personnel.

**Author Response**: Under the material and methods section, we mention the experiments were conducted in a Biosafety Level 3 laboratory. In addition, we added the following text “with the use of appropriate personal protection equipment to prevent transmission of the infection to personnel”.

The authors have also not mentioned the source of the virus. Both these aspects are relevant in case the study leads to further studies of a similar nature looking at any other aspects of the topic/subject matter.

**Author Response**: Under the material and methods section we mention that purified SARS-CoV-2 strains including IC19 (lineage B.1.13), Beta (lineage B.1.351) and Delta (lineage B.1.617.2), were received from Imperial College, London (lines 127-128).

Should you require further information, please do not hesitate to contact me.

I trust that this revision will meet with your approval and look forward to hearing from you.

*Sin cera*

______________________

**Bavesh Kana (PhD)**

Bavesh Kana (PhD)

Professor

Member of the Academy of Science of South Africa (ASSAf)

Member of the Health Professionals Council of South Africa (HPCSA)

University of the Witwatersrand and the National Health Laboratory Service

Johannesburg, South Africa

Tel: +27 11 489 9030 | Fax: +27 11 489 9397

bavesh.kana@nhls.ac.za | http://www.wits.ac.za/cbtbr
